# R-AI-diographers: investigating the perceived impact of artificial intelligence on radiographers' careers, roles, and professional identity in the UK

**DOI:** 10.3389/fdgth.2025.1603511

**Published:** 2025-12-08

**Authors:** Gemma Walsh, Nikolaos Stogiannos, Benard Ohene-Botwe, Kevin McHugh, Alexander Spurge, Ben Potts, Christopher Gibson, Winnie Tam, Chris O’Sullivan, Anton Sheahan Quinsten, Rodrigo Garcia Gorga, Dávid Sipos, Elona Dybeli, Moreno Zanardo, Cláudia Sá dos Reis, Nejc Mekis, Carst Buissink, Andrew England, Charlotte Beardmore, Altino Cunha, Amand H. Goodall, Janice St John-Matthews, Mark McEntee, Yiannis Kyratsis, Christina Malamateniou

**Affiliations:** 1 CRRAG Research Group, Division of Radiography, Department of Allied Health, School of Health and Medical Sciences, City St George’s University of London, London, United Kingdom; 2 Medical Imaging, Spire Healthcare, Washington, United Kingdom; 3 Medical Diagnostic Center, Magnitiki Tomografia Kerkiras, Corfu, Greece; 4 School of Dental, Health & Care Professions, University of Portsmouth, Portsmouth, United Kingdom; 5 Medical Imaging, Portsmouth Hospitals University NHS Trust, Portsmouth, United Kingdom; 6 Radiology, University Hospital Southampton NHS Foundation Trust, Southampton, United Kingdom; 7 Nuclear Medicine Department, Maidstone and Tunbridge Wells NHS Trust, Maidstone, United Kingdom; 8 Institute of Diagnostic and Interventional Radiology and Neuroradiology, University Hospital Essen, Essen, Germany; 9 Nuclear Medicine Service, Hospital Universitari Parc Taulí, Sabadell, Spain; 10 Department of Medical Imaging, Faculty of Health Sciences, University of Pécs, Pécs, Hungary; 11 Department of Medical Technical Specialties, Faculty of Medical Technical Sciences, University of Elbasan “Aleksander Xhuvani”, Elbasan, Albania; 12 Unit of Radiology, IRCCS Policlinico San Donato, San Donato Milanese, Italy; 13 HESAV, Haute école de Santé - Vaud, University of Applied Sciences and Arts Western Switzerland (HES-SO), Lausanne, Switzerland; 14 Medical Imaging and Radiotherapy Department, Faculty of Health Sciences, University of Ljubljana, Ljubliana, Slovenia; 15 European Federation of Radiographer Societies (EFRS), Cumieira, Portugal; 16 University of Applied Sciences, Groningen, Netherlands; 17 Discipline of Medical Imaging and Radiation Therapy, School of Medicine, University College Cork, Cork, Ireland; 18 The Society and College of Radiographers, London, United Kingdom; 19 Bayes Business School, City St Georges, University of London, London, United Kingdom; 20 Visiting Senior Scholar, The Department of Midwifery and Radiography, City St Georges, University of London, London, United Kingdom; 21 Health Services Management & Organisation (HSMO), Erasmus School of Health Policy & Management, Erasmus University Rotterdam, Rotterdam, Netherlands; 22 European Society of Medical Imaging Informatics (EuSoMII), Vienna, Austria

**Keywords:** artificial intelligence (AI), radiographer, radiography, professional identity, clinical roles, AI education, workforce preparedness

## Abstract

**Introduction:**

Artificial Intelligence (AI) is being increasingly integrated into radiography, affecting daily responsibilities and workflows. Most studies focus on AI’s influence on clinical performance or workflows; fewer explore radiographers' perspectives on how AI affects their roles and the profession. This study aims to investigate the perceived impact of AI on radiographers' careers, roles and professional identity in the UK.

**Methods:**

A UK-wide, cross-sectional, online survey including 32 questions was conducted using snowball sampling to gather responses from qualified radiographers and radiography students. The survey gathered data on: (a) demographics, (b) perceived short-term impacts of AI on roles and responsibilities, (c) potential medium-to-long-term impacts, (d) opportunities and threats from AI, and (e) preparedness to work with AI. Overall perceptions (optimism, neutrality, or pessimism) were derived from cumulative answers to a subset of 6 questions.

**Results:**

A total of 322 valid responses were received, showing general optimism about medium-to-long-term impact of AI on careers, roles and professional identity (60.7% optimistic). Most respondents (70.8%) reported no formal AI education or training, with AI education emerging as the top priority for improving preparedness in clinical practice. Concerns centered around the potential deskilling of radiographers and AI inefficiencies. However, 81.2% agreed AI would not replace radiographers in the long term.

**Conclusion:**

Radiographers are broadly optimistic about AI's impact but express concerns about deskilling due to reliance on AI. While their optimism is encouraging for recruitment and retention, there is a clear need for AI-specific education to enhance preparedness to work with AI.

## 1 Introduction

Over the past decade, artificial intelligence (AI), primed by enhanced computational power and the availability of big data, has witnessed a notable surge in applications within healthcare. AI has become increasingly prominent in radiography and radiotherapy (1–3), impacting radiography workflows in different areas, from scheduling patient appointments and guidance on accurate patient positioning, to image optimisation and the facilitation of image interpretation (4, 5). Healthcare systems, like the National Health Service (NHS) in the UK, face an acute shortage of radiologists and radiographers amid rising demands for medical imaging tests and radiotherapy treatment, resulting in unsustainable workloads and a massive exodus of healthcare practitioners in these fields (6). The deployment of AI applications in these settings holds huge potential to sustain and optimise clinical service delivery (7, 8).

Despite its potential and often hyped expectations for its future use, AI is often portrayed negatively in mainstream media (9). Public statements linked to AI include the 2016 statement by Turing award and Nobel prize winner computer scientist, Professor Geoffrey Hinton, who stated that AI had become so effective and efficient, that society could “stop training radiologists within five years” (10). As of October 2024, radiologists are still in high demand, and radiologist training shows no sign of stopping; instead it is ramping up (11). Social media negativity about AI and new regulatory initiatives to tighten the governance framework around AI were seen by some as threats to the pace of AI implementation and its widespread use in clinical practice (12). Furthermore, lack of transparency and a “black-box” culture often result in mistrust or lack of understanding of the technology by the public and clinical professionals (13, 14). Black box culture refers to environments where the under-lying workings of AI tools are not understood by the end-users or those that deploy them (15, 16). Despite these challenges, the implementation of AI into the daily radiography workflow is ongoing, propelled by early findings that show promise in improving efficiency and clinical effectiveness within medical imaging and radiotherapy for the benefit of the patients (17).

With more than 80% of new Food and Drug Administration (FDA) requests in 2023 coming from medical imaging, this field is at the forefront of AI implementation (18, 19). Health Education England (2022) presented an “AI roadmap” reporting on the current AI landscape in healthcare (20). This report found that of 155 healthcare workforce groups, medical roles in “clinical radiology” were the most impacted by AI technologies. Within AI deployment in medical diagnostics, 37% directly impacted roles in clinical radiology. This has inevitably resulted in radiologists and radiographers leading this service transformation (21–23). Being at the forefront of AI-driven change in clinical practice means they are also more vigilant and aware than other healthcare professionals about the imminent changes that AI could bring to their professional role and practices.

When a disruptive new technology such as AI, which is suggested could replace humans, is adopted, professionals might feel threatened. This is because they are often required to reskill and reconfigure their professional roles and identities (24, 25). Professional identity can be described as “the relatively stable and enduring constellation of attributes, beliefs, values, motives and experiences, in terms of which people define themselves in a professional role” (26). Or, as more simply described, the way by which professionals see themselves in terms of who they are, and what they do.

Radiographers' professional identity has traditionally assumed a dyadic nature: (i) one encompassed by the solid command of scientific technology (technological care) and (ii) the other relating to mastering the humane, humanistic work (humanistic care) (27). In all expressions of professional practice, whether this is clinical, educational or research, radiography principles are underpinned by the following key expectations: (1) Promoting and upholding relationships with patients and carers, including making patient safety the primary concern; (2) Maintaining high standards of professionalism and accountability, ensuring patient and public trust in the profession and (3) Upholding technical and professional practice by working within current legal, ethical, professional and governance frameworks (28). Fulfilling these tasks requires radiographers to show excellent command not only of humanistic principles but also demonstrate competency with effectively using the new technology, to acquire and process high-quality images and treat patients expertly.

With the increasing awareness and implementation of AI, it is vital to understand radiographers' experiences, perceptions, and expectations, as key professionals for AI adoption in clinical practice. In particular, the perceived impacts of AI on radiographers' careers, roles, and professional identity needs to be thoroughly explored, to assess how AI integration might reshape their professional landscape, influence job security, and redefine their roles within the healthcare system. This understanding is crucial to ensure that radiographers are adequately prepared and supported in adapting to these changes, thereby maintaining their professional identity and continuing to provide high-quality patient care.

To date, a limited number of studies have examined the perceived impact of artificial intelligence (AI) on radiography careers, professional roles, and identity (2–5, 28). Malamateniou et al. (2), Hardy and Harvey (3), and Lewis et al. (4) investigated these issues through academic commentaries and narrative approaches, offering perspectives on the importance of targeted education and training for radiographers, as well as advocating for collaborative engagement between radiographers and AI systems to promote safe, effective, and ethical implementation and use. Two empirical studies; Rainey et al. (5) in the UK and Abuzaid et al. (28) across the Middle East and India, employed online survey methodologies to assess radiographers' readiness and confidence regarding AI integration. While both studies identified a generally optimistic outlook, they also reported low levels of technical confidence among respondents.

Although existing literature has addressed radiographers' AI education and preparedness, the potential implications for professional identity remain underexplored. The present study seeks to address this gap by examining how AI integration may influence radiographers' roles, responsibilities, and professional identity in the future. It further provides a platform for radiographers to articulate concerns regarding the evolving nature of their work. Given significant developments in the AI landscape since the most recent of these prior studies (2021), and a growing awareness of AI among radiography professionals, this research captures up-to-date perspectives on the anticipated impact of AI on the radiography workforce.

## 2 Materials and methods

### 2.1 Survey governance

Ethical approval (ETH2223-1346) was granted by City St Georges, University of London. Informed consent was obtained from all participants using an online information sheet, and an explicit consent button was embedded on the survey's first page (29). Data collection was anonymous. Minimal demographic data was collected, to ensure meaningful correlations for data analysis. The survey platform does not allow multiple responses from a single IP address (30). To ensure anonymity and confidentiality, the survey was set-up to prevent authors from seeing the IP addresses of respondents. An integrated “back” button and “progress” bar within the survey enhanced participants' navigation, ensuring the authenticity of their responses and maximising completeness rates. Progression to subsequent questions required completion of the earlier questions to minimise the complexity of the analysis.

### 2.2 Design, development and piloting of survey

This was a cross-sectional online survey, adhering to the STROBE and CHERRIES reporting guidelines (31, 32). The study employed a semi-structured questionnaire, consisting of both quantitative (closed) questions and qualitative (open-ended) questions. This was a European-wide survey, with this paper reporting the results from radiographers practicing in the UK. The target population for the survey included students, trainees, apprentices, and qualified radiographers from all areas of practice (clinical, education, research, industry, and policy). As this was an open survey, snowball sampling was used (33).

The survey was developed on Qualtrics (Qualtrics, Provo, Utah, USA) (30). Themes of the survey, and survey question development, were informed by a rapid review of the literature (34), and early discussions in focus groups and interviews with different radiography experts in Europe. Various iterations of the survey were created based on these discussions and then collectively reviewed by the project leads, who have expertise in healthcare leadership, radiography, and professional identity. Two review rounds of the survey by the project team followed, ensuring content validity (35).

Furthermore, the survey was piloted on a small sample size (*n* = 12) of radiographers to assess the internal consistency of the questionnaire. This was conducted immediately before the release of the survey. The responses collected from the pilot study were analysed using Cronbach's alpha reliability tool (36). Three constructs of the questionnaire focused on radiographers' perspectives on the short (Construct A), medium (Construct B), and long-term (Construct C) impacts of AI on the profession were tested for internal consistency.

### 2.3 Data collection instrument

The data collection instrument consisted of an online survey with 32 questions, including both open-ended and closed-type questions (provided as supplementary material). Questions explored the following areas: (a) participant demographics, including the self-assessed level of AI knowledge, education, and experience (11 questions), (b) radiographers' perceptions of the short-term impact of AI (12 questions) (c) medium-to-long-term expected impact of AI on radiographer roles, responsibilities and professional identity (4 questions), (d) perceived opportunities and threats of AI implementation (4 questions) and (e) the level of preparedness of radiographers to work with AI (1 question).

### 2.4 Recruitment and eligibility of participants

Recruitment was voluntary, and no incentives were offered. Data collection occurred between June 1st and August 31st, 2023. To maximise recruitment, the study co-authors sent out electronic reminders once monthly.

This survey was endorsed by the European Federation of Radiographer Societies (EFRS) (37), who kindly shared it amongst their member societies and through internal and external communication channels. All co-authors also electronically distributed to their professional networks in academia, research, policy, and practice in the UK via personal email and social media (LinkedIn®, X®, and Facebook ®) for maximum coverage. Face-to-face recruitment, utilising a QR code for the online survey, also took place within the Research Hub at the UK Imaging and Oncology Congress (UKIO) (38) in June 2023.

Eligible participants included those aged 18 and above, studying or practicing radiography or radiotherapy in the UK in any capacity. Anyone trained as a radiographer was eligible to join the study, including those working in academia, industry, or retired.

### 2.5 Data analysis

As required, descriptive and inferential statistics were employed for quantitative data, and graphs were plotted to illustrate comparisons and correlations. Non-parametric statistics were used to compare between groups (Mann–Whitney *U* test) (39) and explore correlations (Spearman's rank) (40). Comparisons, where eligible, were run between the groups for: (a) age range (above and below 35 years of age), (b) gender, (c) years of experience, (d) highest academic qualification and (e) level of AI knowledge, after appropriate thresholds were defined. A threshold example, decided upon team consensus, included a cut-off point of over 10 years to differentiate the more experienced professionals from the less experienced ones. Correlations were run for all Likert scale questions for the same variables. Before the inferential analysis, all relevant variables were recoded into numerical values for statistical analysis. For Likert scale questions, 1 was assigned to the most negative response and 5 to the most positive. Answers of “strongly agree” and “agree” were grouped (as were “strongly disagree” and “disagree”) to help to understand overall trends of agreement or disagreement with a given statement. A *p*-value of less than 0.05 was used to denote statistical significance.

A “sense of optimism” for AI with respect to the short-term and medium-to-long-term effects on radiographer roles and careers was calculated. Table 1 details the six questions and the respective answer options to assess professional optimism/pessimism with AI. Percentages were calculated with respect to cumulative optimistic answers.

**Table 1:** Illustration of a subset of questions used to assess radiographer's overall optimism/pessimism with AI.

Short-Term Impacts of AI Implementation					
Question	“Despite the advancement of AI, image quality and treatment quality will remain the responsibility of the radiographers.”				
Answers	**Strongly Agree**	**Agree**	**Neither Agree nor Disagree**	**Disagree**	**Strongly Disagree**
Question	“Medical image and treatment quality will become the responsibility of AI (with appropriate quality assurance checks).”				
Answers	**Strongly Agree**	**Agree**	**Neither Agree nor Disagree**	**Disagree**	**Strongly Disagree**
Question	“With regards to AI advancements, radiographer job and career opportunities will.”				
Answers	**Increase**	**Remain the Same**		**Decrease**	
Medium-to-long-Term Impacts of AI Implementation					
Question	“AI will only ever assist radiographers, never replace them.”				
Answers	**Strongly Agree**	**Agree**	**Neither Agree nor Disagree**	**Disagree**	**Strongly Disagree**
Question	“With time, AI will ultimately replace radiographers.”				
Answers	**Strongly Agree**	**Agree**	**Neither Agree nor Disagree**	**Disagree**	**Strongly Disagree**
Question	“Radiographers will evolve with AI, and roles and professional identity may be quite different from today.”				
Answers	**Strongly Agree**	**Agree**	**Neither Agree nor Disagree**	**Disagree**	**Strongly Disagree**

Participants who gave collective “positive” answers highlighted in green, in the two areas of short and medium-to-long-term impacts, were deemed to be optimistic for the future of radiographer roles and professional identity with the implementation of AI. Participants who gave collective “negative” answers (highlighted red) were deemed as pessimistic, whereas those giving collective neutral answers (highlighted orange) were considered neutral overall.

Each respondent was assigned a unique identifier (e.g., R1), for their open-ended responses. All data in the open-ended questions was coded, categorised and summarised into themes, using qualitative content analysis (41). Content analysis was performed manually, identifying key codes and categories and then reducing the categories into themes. This process was recorded using Microsoft Excel. The two primary authors conducted independent, blinded analyses, ensuring neither was aware of the other's findings. The study's primary investigator subsequently reviewed the final analysis for accuracy and consistency. When reporting qualitative quotes from open-ended questions, demographic details, including gender, age, and self-reported level of AI knowledge have been provided to contextualise responses in alignment with statistically significant results and discussion points.

## 3 Results

### 3.1 Survey robustness

The Cronbach's alpha results indicated satisfactory internal consistency within each construct. Construct A yielded a Cronbach's alpha (*α*) of 0.754, while Construct B demonstrated slightly higher internal consistency (*α* = 0.792). The lowest internal consistency (*α* = 0.739) was observed in Construct C, though it was still within the acceptable range of reliability according to Bland (42). Collectively, the data instrument exhibited acceptable internal consistency (*α* = 0.780) before its application in the main study.

### 3.2 Demographics

There were 322 valid responses from radiographers in the UK, including students (14.9% *n* = 48), practitioners (82.8% *n* = 267), retired (1.6% *n* = 5) and others (0.6% *n* = 2) (Table 2). The majority of respondents were female (66%) and trained as diagnostic radiographers (76.7%). The highest percentage of respondents had 11–20 years' experience as a radiographer (29.2%), with a bachelor's degree (or DCR equivalent) as their highest qualification (32.9%) and worked in a public hospital (71.7%). The majority also had a self-reported “basic” knowledge of AI (55.3%) and had never used AI to their knowledge (56.8%) (Table 3).

**Table 2:** Summary of respondent demographics.

Demographics	Variables	Percentage (%)	Frequency (*n*)
Gender	Female	66	211
	Male	32	104
	Non-Binary	1	4
	Prefer not to say	1	3
	Total	100	**322**
Age	18–25	12.4	40
	26–35	34.8	112
	36–45	24.8	80
	46–55	19.6	63
	56–65	6.5	21
	>65	1.9	6
	Total	100	**322**
Radiography Experience (years)	0–2	12.4	40
	3–5	14.3	46
	6–10	15.2	49
	11–20	29.2	94
	>20	28.0	90
	Not practicing	0.3	1
	Retired	0.6	2
	Total	100	**322**
Main Current Role	Undergraduate Student Radiographer	14.9	48
	Apprentice Radiographer	0.3	1
	Assistant Practitioner Radiographer	0.0	0
	Clinical Radiographer	41.3	133
	Research Radiographer	4.0	13
	Advanced Practitioner Radiographer	12.4	40
	Consultant Radiographer	0.9	3
	Radiology Manager	6.8	22
	Clinical Academic	5.6	18
	Academic in Radiography- teaching only	5.6	18
	Academic in Radiography- teaching + research	5.6	18
	PhD Student in Radiography	0.3	1
	Retired Radiographer	1.6	5
	Other	0.6	2
	Total	100	**322**
Radiographer Specialty	Diagnostic Radiographer	76.7	247
	Therapeutic Radiographer	14.9	48
	Both Diagnostic + Therapeutic Radiographer	1.2	4
	Nuclear Medicine Radiographer	1.9	6
	Sonographer	5.0	16
	Other	0.3	1
	Total	100	**322**
Highest Academic Qualification	Still studying as an undergraduate student	11.2	36
	BSc (or DCR or equivalent)	32.9	106
	Post Graduate Certificate	15.8	51
	Post Graduate Diploma	13.4	43
	Master's (or MBA/equivalent)	23.0	74
	PhD/Professional Doctorate	3.7	12
	Total	100	322
Main work setting	Public Hospital	71.7	231
	Private Hospital/Centre	8.1	26
	Research Centre/Institute/Facility	1.6	5
	Mobile Unit	6.5	21
	Not working in a clinical setting	12.1	39
	Total	100	**322**

Percentages have been reported to one decimal place.

**Table 3:** Self-reported radiographer level of AI knowledge and the frequency of AI use.

Question	Answer	Percentage (%)	Frequency (*n*)
AI Knowledge	Never heard of AI	5.0	16
	Basic	55.3	178
	Intermediate	32.9	106
	Advanced	6.2	20
	Expert	0.6	2
	Total	100	**322**
AI Experience/Use	Never used	56.8	183
	Occasional	19.6	63
	Daily	14.3	46
	Involved in Research	9.3	30
	Total	100	**322**

### 3.3 Type of AI education and training for UK radiographer respondents

Quantitative and qualitative data were collected. The number of valid responses (*N*) varied across the results. Reductions in *N* were attributable to standard participant attrition, while increases reflected instances where multiple responses were submitted by the same individual among the total sample of 322 participants. Radiographers were allowed to select more than one option when answering questions about AI education and training in which they had participated. This resulted in 408 quantitative responses, of which 38% have never received any AI training. This increased to 70.8% with no formal AI education or training once those who had been self-taught through personal study had been included (32.8%) (Table 4). A total of 81 respondents opted to leave qualitative comments regarding the AI education they had participated in, resulting in 7 categories and 89 different examples of AI educational provisions (*n* = 89) (Table 4).

**Table 4:** Summary of AI education and training undertaken by UK radiographers.

Category	Percentage (%)	Quantitative frequency (*n* = 408)	Examples of top answers (total number of qualitative occurrences *n* = 89)
None	38	155	Not Applicable
Self-taught-personal-reading	32.8	134	None specified.
CPD Education by Company	8.1	33	*General Electric (GE) (n* *=* *3)* *ProSoma (n* *=* *2)* *Equally; C-RAD, MOSAIQ (Elekta), Guerbet, Coursera, Siemens, Philips, Annalise.ai, Brainomix (n* *=* *1 each)*
Undergraduate Level at University	4.9	20	*General Curriculum (n* *=* *9)* *Study outside of Radiography (n* *=* *3)* *AI Dissertation Topic (n* *=* *1)*
CPD Education by Professional Society	4.7	19	*Society of Radiographers (SoR) (n* *=* *4)* *British Institute of Radiology (BiR) (n* *=* *3)* *Equally; Unspecified MRI Societies, Health Data Research UK (HDRUK), National Institute of Health Research (NIHR), Institute of Physics & Engineering in Medicine (IPEM), Quality Assurance Agency (QAA), Staff and Educational Development Association (SEDA) (n* *=* *1 each)*
Others	4.4	18	*Unspecified CPD (n* *=* *8)* *Conferences (UKIO & BNMS) (n* *=* *7)* *Research and Development Involvement (n* *=* *5)* *Clinical Training & Discussions (n* *=* *2)*
Postgraduate Level at University	3.7	15	*General Curriculum (n* *=* *11)* *AI Dissertation Topic (n* *=* *8)* *Study Outside of Radiography (n* *=* *1)*
CPD Education at University	3.4	14	*City, University of London- Introduction to AI for Radiographers Module (n* *=* *4)* *Unspecified CPD (n* *=* *4)*
Total	100	**408**	89

### 3.4 Perceived short-term effect of AI implementation on radiographer roles, careers and professional identity

**Figure 1:**
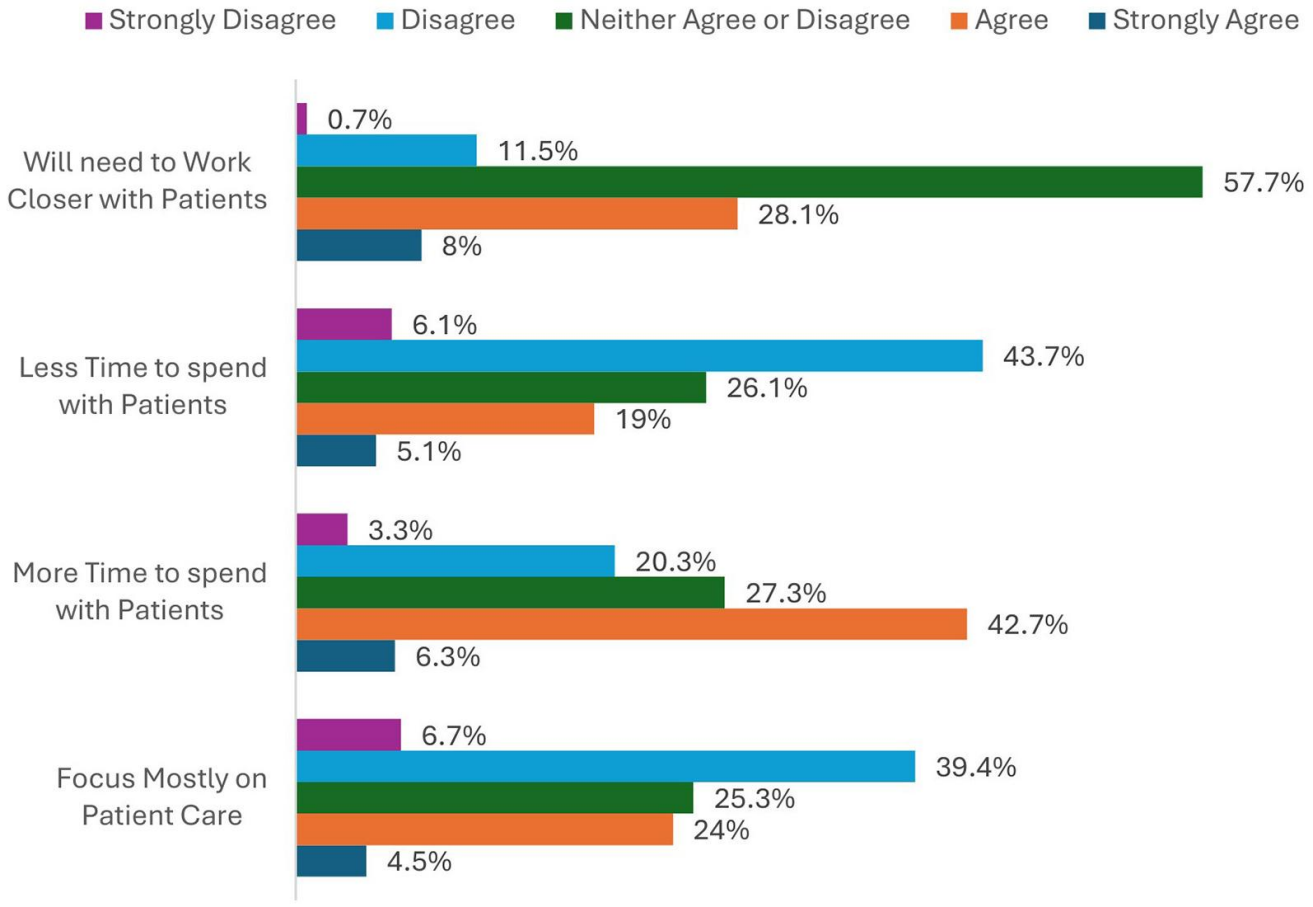
The percentages of respondents when asked of their agreement or disagreement with statements surrounding patient care.

**Figure 2:**
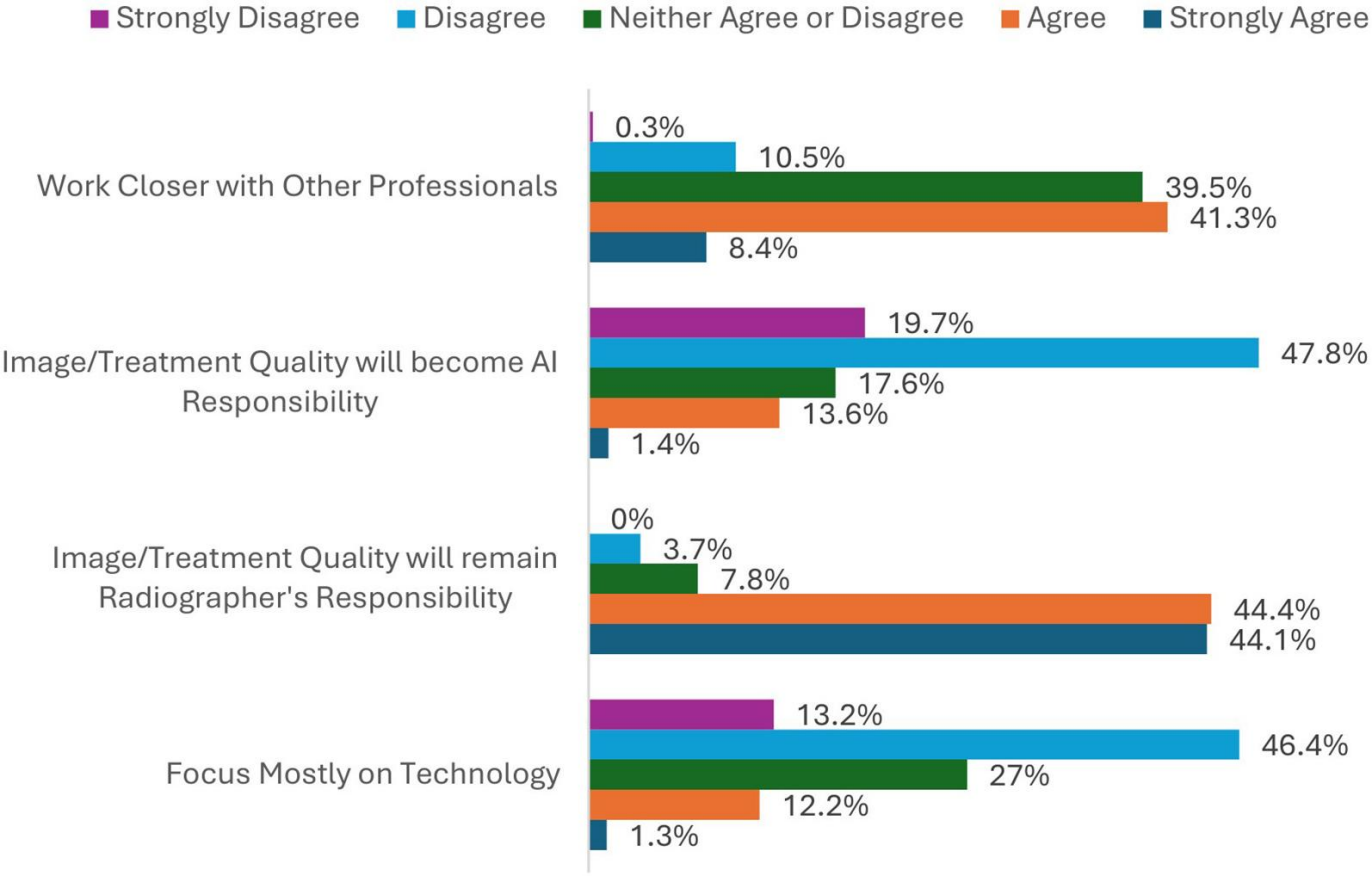
The percentages of respondents when asked about their agreement or disagreement with statements surrounding technology.

In the 5-point Likert scale questions assessing the level of agreement participants had with statements about the future of their patient-centered care skills and technological skills, the largest proportion of respondents believed that with the implementation of AI the future role of the radiographer will not focus mostly on patient care (46.1% disagreement) (Figure 1). However, the future role will provide more time with patients (49% agreement). Most (51.7%) were neutral on whether radiographers will need to work closer with patients. With regards to their technology-related skills (Figure 2), radiographers disagreed that they would become more technology-focused (59.6%); whereas the majority felt that image and treatment quality would remain the responsibility of radiographers, and not AI (88.5% agreement).

**Figure 3:**
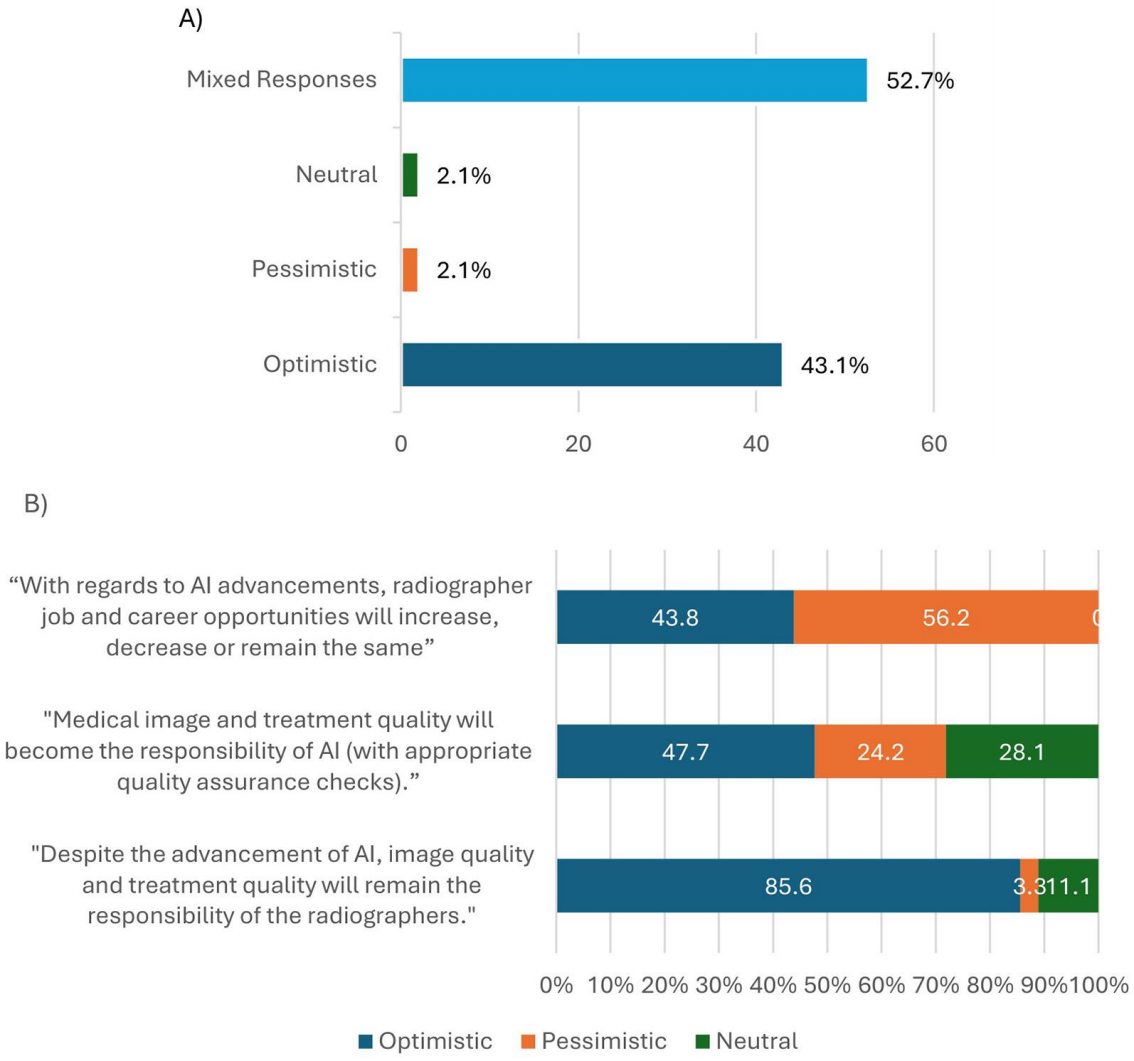
**(A)** Percentages of respondents perceived as optimistic, pessimistic, neutral, or having given mixed responses, to a subset of questions exploring the perceived short-term impact of AI on radiographer job roles and professional identity (Table 1). **(B)** Breakdown of the answers given to each sub-set question by those who gave mixed responses in A (52.7%). Optimistic responses were the highest percentage for each individual question, except when asked about future job and career opportunities with the slight majority giving the pessimistic answer of “decrease” (56.2% *n* = 86).

A total of 295 radiographers answered all three sub-set of questions used to assess short-term perceptions of radiographer roles and responsibilities (Table 1). For the short-term impact of AI implementation, 43.1% answered collectively “optimistically” to all three questions, with 2.1% answering both collectively pessimistic and collectively neutral (Figure 3). There was a high percentage of mixed responses (52.7%). Figure 3 provides a breakdown of these mixed responses.

**Figure 4:**
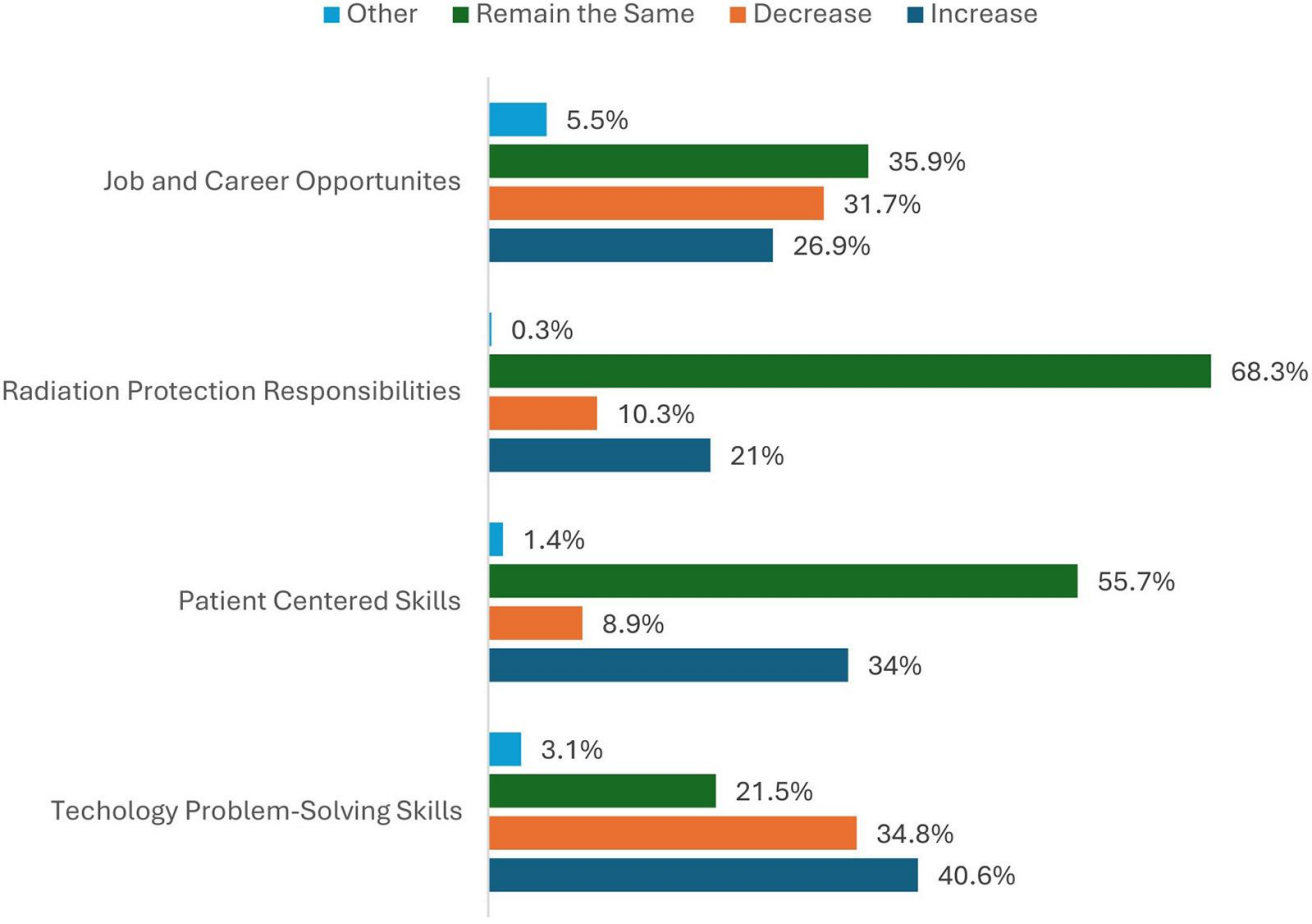
The percentage of respondents that answered “increase”, “decrease”, “remain the same” or “other” to questions relating to changes on radiographer roles and practices.

Participants were asked how radiography and radiotherapy practices would change with the implementation of AI (Figure 4). The highest percentage of respondents reported that technology competencies will increase with the implementation of AI (40.6%). Still, patient-centered care skills will remain the same (55.7%) along with their radiation protection responsibilities (68.3%). Participants remained undecided about whether job and career opportunities will change, with 26.9% thinking they will increase, 31.7% decreasing, and 35.9% believing they will remain the same. Of the 5.5% who answered other, the main qualitative theme was “new opportunities”. Participant R-86 (female, aged >35 years, basic AI knowledge) wrote, “*I believe that there will be many different opportunities. Change rather than increase/decrease*.”

### 3.5 Perceived medium to long-term impacts of AI implementation on radiographer roles, careers and professional identity

**Figure 5:**
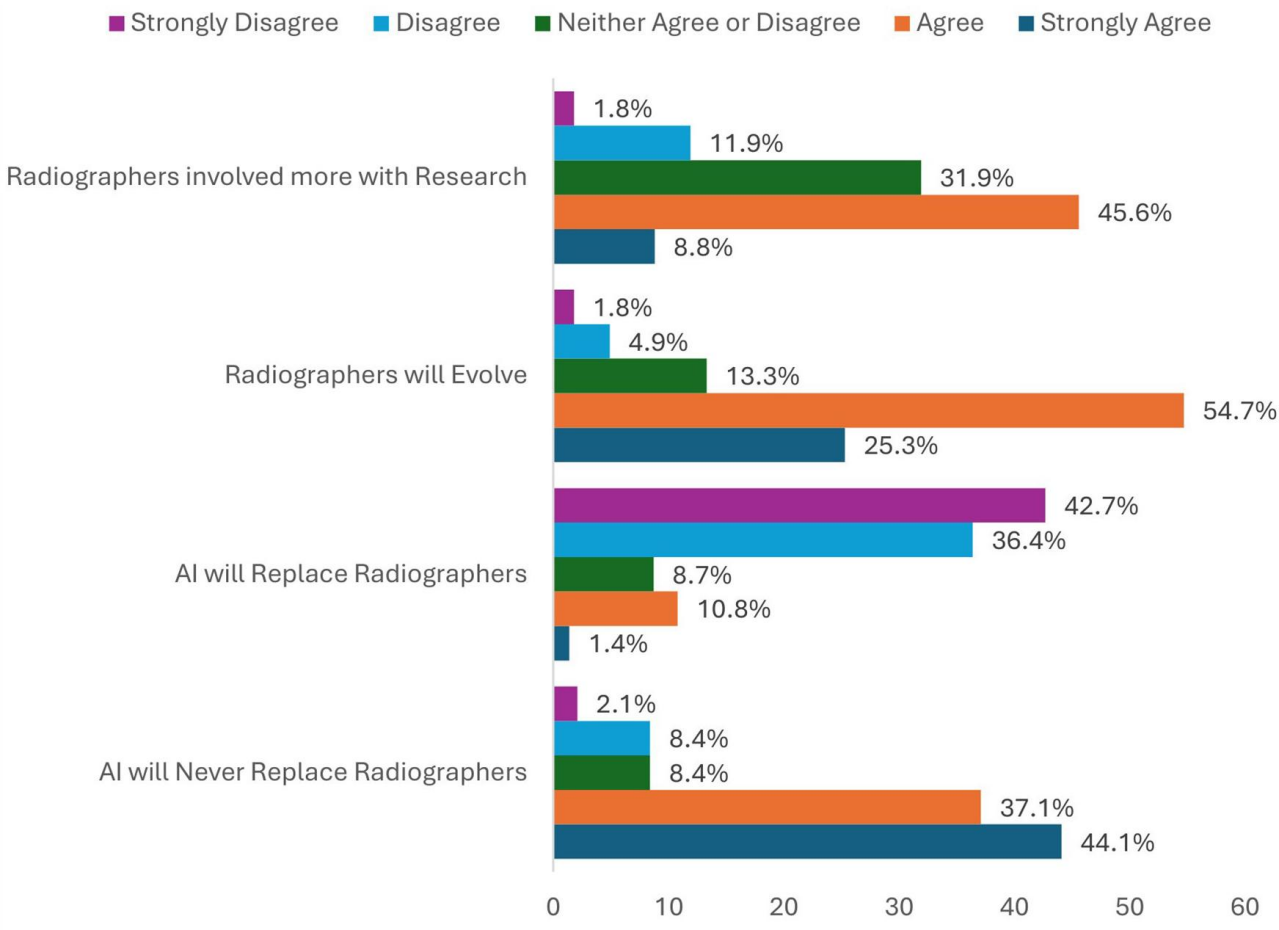
Respondents agreed with statements surrounding the medium-to-long term impacts of AI implementation on radiographer roles, responsibilities, and professional identity.

Reassuringly, the majority of radiographers believed “AI will only ever assist radiographers, never replace them” (81.2% agreement) however they also agreed that “radiographers will evolve with AI, and roles and professional identity may be quite different from today” (80% agreement) (Figure 5).

**Figure 6:**
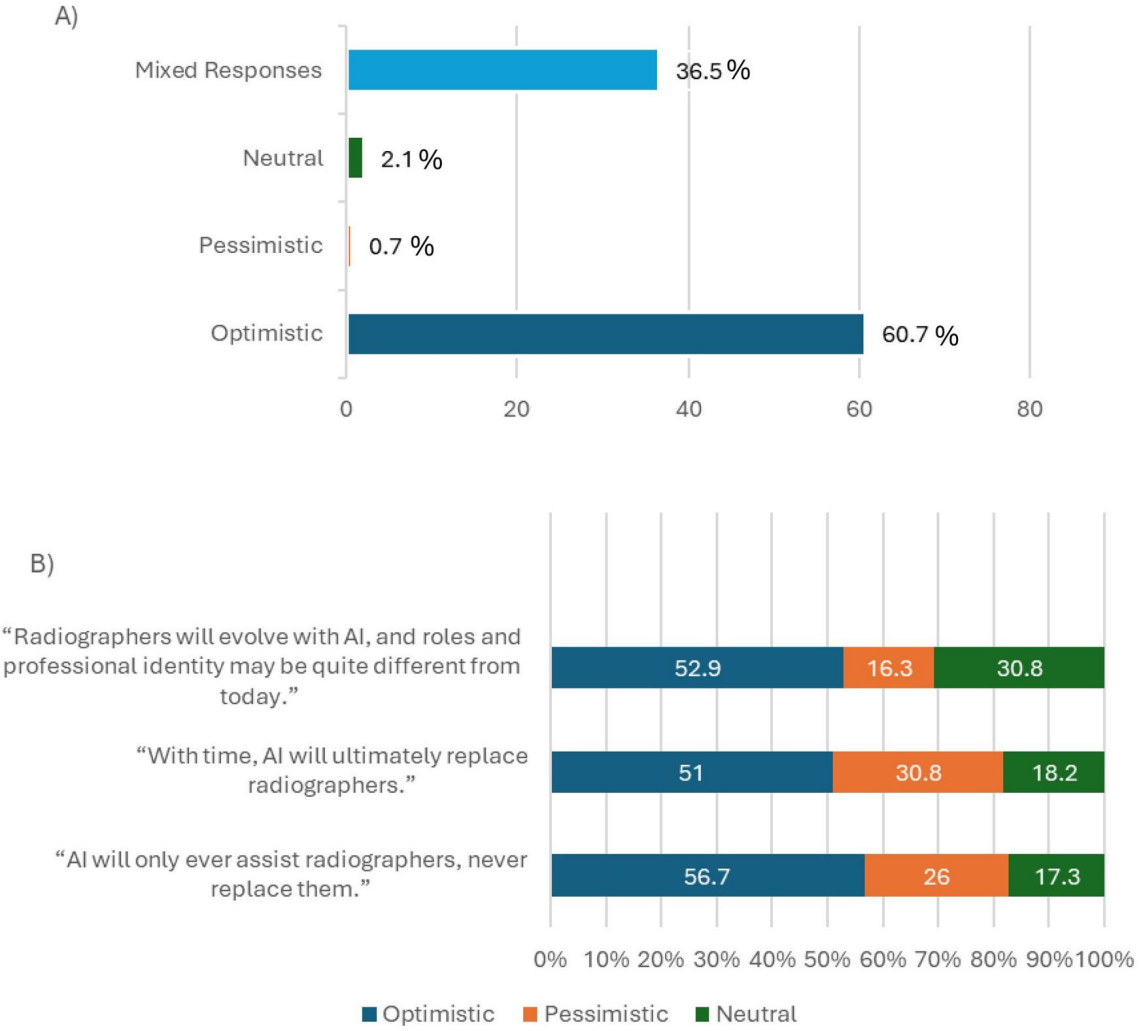
**(A)** Percentages of respondents perceived as optimistic, pessimistic, neutral, or having given mixed responses, to a subset of questions exploring the perceived medium-to-long-term impact of AI on radiographer job roles and professional identity (Table 1). **(B)** Breakdown of the answers given to each sub-set question by those who gave mixed responses in A (36.5%). Optimistic responses were the highest percentage for each question.

In total 285 respondents answered all three sub-set questions to assess overall perceptions (optimism/pessimism) for the longevity of the radiographer role (Table 1). Of these 285, 173 (60.7%) were considered fully optimistic for the long-term future of their profession. From the remaining 39.3%, 0.7% (*n* = 2) gave collective negative answers, 2.1% (*n* = 6) were impartial, and 36.5% gave mixed responses. Figure 6 provides a breakdown of these mixed responses.

### 3.6 Inferential statistics

Statistically significant inferential statistics are summarised in Table 5. Regarding patient care skills, respondents aged >35 were more likely to agree that with AI implementation, radiographers will have more time to spend with patients, than the 18–35 year-old group (*p* = 0.006). This is also true for those with postgraduate studies compared to those with undergraduate studies (*p* = 0.011). This notion is also reinforced by radiographers with more experience (greater than ten years), agreeing that with AI implementation, “radiographers will be required to focus mainly on patient care and less on technology” (*p* = 0.023) compared to those that have less than ten years' experience.

**Table 5:** Statistically significant differences between groups within variables (Mann–Whitney *U* test).

Variables	Categories of respondents	Mean score on a scale of 1 (strongly disagree)-5 (strongly agree)	*p*-value	Effect size according to Cohen's classification
Radiographers will have less time to spend with patients due to a potential increase in workload	18–35 years old	2.83	0.011	0.148 (small effect)
	>35 years old	2.59		
Radiographers will have more time to spend with patients due to faster workflow	18–35 years old	2.74	0.006	0.159 (small effect)
	>35 years old	3.06		
Radiographers will be more involved in research and development than their current role.	18–35 years old	3.57	0.047	0.117 (small effect)
	>35 years old	3.40		
Radiographers will be required to focus mostly on technology and less on patient-related responsibilities	None to intermediate AI knowledge	2.57	0.030	0.215 (small effect)
	Advanced and experts	3.09		
Radiographers will need to work closer with the patients	None to intermediate AI knowledge	3.28	0.010	0.151 (small effect)
	Advanced and experts	3.79		
Radiographers will be required to focus mostly on patient care and be less involved in technology	Less experienced	2.83	0.023	0.151 (small effect)
	More experienced	3.06		
Radiographers will evolve with AI, and roles and professional identity may be quite different from today	Male radiographers	4.09	0.026	0.133 (small effect)
	Female radiographers	3.90		
Radiographers will need to work closer with the patients	Male radiographers	3.50	0.010	0.153 (small effect)
	Female radiographers	3.21		
Medical image and treatment quality will become the responsibility of AI	Undergraduate studies only	3.23	0.019	0.136 (small effect)
	Postgraduate studies	3.53		
Despite the advancement of AI, image quality and treatment quality will remain the responsibility of the radiographers	Less experienced	3.24	0.013	0.144 (small effect)
	More experienced	2.95		
Radiographers will have more time to spend with patients due to faster workflow	Undergraduate studies only	2.73	0.011	0.146 (small effect)
	Postgraduate studies	3.04		

A *p*-value of less than 0.05 was used to denote statistical significance.

Regarding radiographer competencies, radiographers aged >35 years of age are less likely to believe radiographers will become more involved in research and development with AI implementation than their younger colleagues (*p* = 0.047). Male radiographers were more likely to agree with the statement “radiographers will evolve with AI, and that roles and professional identity may be different from today” compared to females (*p* = 0.026).

### 3.7 Opportunities and threats of AI implementation on radiographer roles, responsibilities and professional identity, and radiographer preparedness to work with AI

Content analysis was used to generate themes from open-ended qualitative questions surrounding the perceived opportunities and threats generated by AI implementation (4 questions) and a single question investigating the level of preparedness of Radiographers to work with AI (Table 6). The top emerging themes were “AI Education and Training” (*n* = 133), emerging in response to the question, “*What do you feel you mostly need to be better prepared to work with AI in your practice?*” and “Impact on Professional Skills” (*n* = 106), which featured prominently when respondents considered, “*What concerns you more as AI comes into Radiography Practice?*”

**Table 6:** The strongest emerging themes, and associated quotes from the four questions investigating the perceived opportunities and threats of AI to the radiographer profession, and radiographers' preparedness to work with AI.

Theme	Example quotes
Perceived Opportunities and Threats	
*“What are you looking forward to as AI comes into daily Radiographer Practice?”*	
a. Enhanced Workflow Efficiency (*n* = 64)	“Faster working practices” R-114 (male >35 years, intermediate AI knowledge). “Less repetitive work” R-137 (female, aged >35 years, intermediate AI knowledge). “AI will play a massive role in vetting requests, protocol selection, allocation of studies to the correct reporting entity.” R-111 (male, aged >35 years, basic AI knowledge).
b. Enhanced Patient Care/Outcome (*n* = 55)	“The equipment we use becoming faster, less prone to errors and making the patient experience more comfortable” R-79 (female, age 18–35 years, Basic AI knowledge). “A more rigorous quality of diagnosis for patients, as when reporting on images AI can often detect things that can be missed by the human eye”. R-130 (female, aged >35 years, basic AI knowledge). “Reduced scanning time allowing for better patient experience and increased patient interaction (MRI).” R-12 (male, aged >35, intermediate AI knowledge).
*“What concerns you more as AI comes into Radiography Practice?”*	
a. Impact on Professional Skills (*n* = 106)	“People not being trained to use their own initiative and becoming dependent on AI without the knowledge or skills to realise when something is incorrect or needs to be changed” R-265 (female, aged >35 years, basic AI knowledge). “Readers may rely on AI and miss obvious abnormalities, assuming the AI is correct” R-129 (female, aged >35 years, intermediate AI knowledge). “I am concerned that decisions may be made about AI that affect radiographers without their involvement” R-263 (non-binary, aged >35 years, basic AI knowledge).
b. AI Efficiency (*n* = 28)	“AI efficiencies being overstated and subsequent reductions in the radiography workforce” R- 273 (male, aged >35 years, advanced AI knowledge). “Increases [more] chance for technology to disrupt patient pathways & ultimately breaks in treatment due to breakdowns’’ R- 274 (female, aged 18–35 years, basic AI knowledge). “Validation of the AI solutions, my understanding is that many solutions have been effective in a test environment, and I would like to understand how these perform in real life conditions to ensure they cause no detriment’’.R-22 (female, aged >35 years, basic AI knowledge).
*“How do you think Radiographer roles and responsibilities will change, if at all, with the introduction of AI?”*	
a. Role Enhancement (*n* = 82)	“…I also expect more roles and advanced/specialist roles and pathways can open up for radiographers” R-287 (male, aged >35 years, intermediate AI knowledge). “Radiographers are going to be needed in AI education, research and policy-making” R-99 (male, aged 18–35 years, expert AI knowledge). “Expect greater role in QA, validation and development of AI solutions.”R-12 (male, aged >35 years, intermediate AI knowledge).
b. Status Loss (*n* = 26)	“Radiographers will no longer be professionals but technicians or healthcare assistants” R-4 (female, aged >35 years, intermediate AI knowledge). “I think it will become a more technical, supervisory post, overseeing AI, rather than working with it”. R-44 (female, aged >35 years, intermediate AI knowledge). “…Seen even more as ‘button pushers’…”R-215 (female, aged >35 years, basic AI knowledge).
*“What do you expect the impact of AI on radiographer professional autonomy will be?”*	
a. Positive Impact—increased autonomy and confidence (*n* = 61)	“There will be more professional autonomy if we choose to maximise the potential that AI poses” R-14 (female, aged >35 years, advanced AI knowledge). “Radiographers will have increased autonomy and more confidence in that autonomy” R-278 (male, aged 18–35 years, intermediate AI knowledge). “Greater autonomy as radiographers can be the key leaders of radiology with the aid of AI technology” R-283 (male, aged 36–45, intermediate AI knowledge).
b. Negative Impact—threat of becoming technology dependent (*n* = 42)	“I believe radiographers will have less freedom in the decision of a patient care plan” R-69 (female, aged 18–35 years, basic AI knowledge). “… there is potential for perception of autonomy to slip, and these radiographers may become reliant on following a set procedure without knowing the reasoning behind it.” R-16 (non-binary, aged 18–35 years, intermediate AI knowledge). “Reduced opportunities for professional autonomy”R-301 (male, aged 18–35 years, basic AI knowledge).
Preparedness to work with AI	
“What do you feel you mostly need to be better prepared to work with AI in your practice?”	
AI Education and Training (*n* = 133)	“More educational provision. At undergraduate level, it is nearly non-existent. By educating students on AI and its applications in practice, fear and misunderstanding can be dispelled at an early stage” R-201 (male, aged 18–35 years, intermediate AI knowledge). “More education, I'd like to learn much more about it but don't know where is best to educate myself.”R-310 (female, aged 18–35 years, basic AI knowledge). “A greater understanding of how AI will integrate with radiography.” R-301 (male, aged 18–35 years, basic AI knowledge).
AI Trustworthiness (*n* = 6)	“…reassurance that the technology is reliable and accurate” R-30 (female, aged >35 years, basic AI knowledge). “More research into pedagogy around using AI” R-83 (male, aged >35 years, Advanced AI knowledge). “Full trust in the AI systems and how they work.” R-282 (female, aged >35 years, intermediate AI knowledge).

Each respondent was assigned a unique identifier (e.g., R1), which follows each quote. Demographic details, including gender, age, and self-reported level of AI knowledge are provided to contextualise responses in alignment with statistically significant results and discussion points.

Quantitative findings, particularly those reaching statistical significance or representing the most prevalent responses, will be explored in greater detail within the discussion. Where applicable, qualitative data will be used to support quantitative results, and the most salient emergent themes will inform the overall interpretation and conclusions.

## 4 Discussion

This study explored radiographer perspectives around future careers, roles and professional identity, and was necessary for guiding the future development of key competencies, job specifications, designing customised educational provisions, and revising policy and practice for radiographers. The results of this study could also impact the profession's underlying scope of practice (and underpinning education and training) at pre-registration and post-registration levels.

### 4.1 Radiographer role duality

Different aspects of this project explored the potential impact of AI implementation on the duality of the radiographer role, encompassing command of technology whilst mastering patient care. When asked whether AI implementation will result in radiographers having a stronger patient-centered focus (Figure 1) vs. a stronger technology focus (Figure 2), radiographers disagreed with both (46.1% and 59.6% respectively). Radiographers value their autonomy and the duality of their role, which underpin their professional identity. Literature relating to organisation studies infers that the dynamics of professional identity are underpinned by a concern for professional autonomy and a commitment to professional values (43, 44). Clinicians in particular have been shown to fiercely resist change which undermines their independence or interventions that could compromise patient welfare (45). Radiographers are used to balancing technology and patient care, demonstrating a unique and admirable attribute of digital resilience over many technological advancements the last few decades. This balance seems to be integral to their role, sense of professional identity, and job satisfaction, an attribute they will resist giving up in the future (27).

However, 40.6% of radiographers agreed that technology problem-solving skills are likely to increase in importance for the profession in the future, acknowledging that some specific areas of this duality will need to evolve with AI implementation. Digital skill competency building and AI computational literacy have been highlighted in this paper as key to workforce preparedness (Table 6), aligning with recommendations in the Topol review (46) and findings from previous studies on the use of AI by radiographers and other professionals (5, 34, 47). Upskilling is vital for radiographers to safeguard their roles, but also for patient safety and optimal patient care.

Specific aspects of radiographer roles and responsibilities were further highlighted when exploring the differences in answers by different cohorts of respondents. Most statistically significant findings surrounded patient care whilst touching upon radiographer technology skills and radiographer's overall role and responsibilities (Table 5). Patient care is a key focus of radiographers when discussing the impact of AI.

### 4.2 Demographic differences

#### 4.2.1 Gender discrepancies

Male radiographers were more likely to agree that “radiographers will evolve with AI, and that roles and professional identity may be different from today” compared to female ones (*p* = 0.026). Male respondent R-287 (aged >35 years, intermediate AI knowledge) supports this by suggesting the radiographer role could evolve to include “*advanced/specialist roles and pathways*” for radiographers (Table 6). In response to the statement, “Radiographers will need to work closer with patients”, females exhibited a significantly lower agreement compared to males.

Given this study's aim to examine radiographers' perceptions of the potential impact of AI on the future of the profession, previous literature would suggest gender-based differences were expected to emerge. Previous research suggests that females, when compared to males, exhibit greater skepticism regarding the use, knowledge and adoption of AI (48, 49). This may manifest in female radiographers as reduced expectations for role evolution in response to AI and lower agreement with proposed changes, such as increased patient-facing responsibilities.

Such exhibited caution may be attributed to disparities in training exposure. Training exposure can take the form of formal training or as hands-on experience using AI. Adequate training is often associated with reduced apprehension towards new technologies including AI, and limited exposure may contribute to a heightened sense of uncertainty, fear and distrust. A lack of training exposure could cause female radiographers to potentially report lower preparedness for engaging with AI technologies. Additionally, women are underrepresented in leadership roles related to technological innovation, which may influence their openness to accepting and adopting new technologies (50, 51). Previous findings indicate that female radiographers tend to express lower levels of confidence in using AI technologies compared to their male counterparts (5).

#### 4.2.2 Age and experience

A significant difference was observed between younger (18–35 years old) and older radiographers (>35 years old) regarding the predicted time radiographers will spend with patients in the future (*p* = 0.01 and *p* = 0.006). Older radiographers (>35 years) were more likely to believe they will have more time with patients due to faster workflows (mean agreement score = 3.06) as suggested by respondent R-12, (male, aged >35, intermediate AI knowledge), quoting “*Reduced scanning time allowing for better patient experience and increased patient interaction (MRI)*” when asked what they're most looking forward to with the integration of AI (Table 6). This may be because younger radiographers, exposed to rapid technological advances in recent years, have yet to experience increased patient interaction time. Younger radiographers, being more accustomed to technology (49), may trust it to enhance efficiency, resulting in shorter appointments (52). This may also indicate concerns among older radiographers (>35 years) about current patient interaction time, reflecting hope that AI implementation will free up more time for patient care. Previous publications suggest the potential of AI to speed up radiology work flows (53–55), but how this “extra” time gets used is at the discretion of policy makers and managers. Some may choose to lengthen appointment times, allowing for increased patient care and interaction, whereas others may decrease appointment times but offer additional breaks or administrative flexibility to staff. Younger radiographers may believe this additional time will not be used for increased patient interaction, but will be used for increased patient through-put. These ideas amplify the importance of keeping a reflective lens on AI integration and not to not consider it solely a technological challenge, keeping trust, empathy, and human connection central pillars of healthcare practice (46).

Radiographers with over 10 years of experience and those with postgraduate qualifications were more likely to agree that AI will assume responsibility for image and treatment quality. This may suggest a knowledge gap, as those with over 10 years of experience may not have been exposed to AI during their undergraduate studies, potentially leading to concerns about AI taking over key responsibilities. As only 3.7% of respondents received AI education at the postgraduate level, specialised postgraduate training in AI could reduce this fear and better equip experienced radiographers to work confidently with AI.

There appears to be a correlation between professional maturity (whether through age, education, or experience) and an appreciation for the need to work more closely with patients despite technological advances. More mature professionals may be less captivated by new technologies and recognise that the patient remains at the core of all care, with technology serving as a tool to support this human-centered focus.

#### 4.2.3 AI knowledge

Those with a self-proclaimed “none to intermediate” knowledge of AI are more likely to disagree that “*Radiographers will be required to focus mostly on technology and less on patient-related responsibilities”* compared to those with “advanced and expert knowledge” of AI, who remain more neutral to this statement (*p* = 0.03). This may be explained by those with more AI knowledge and understanding of AI implementation, and that the healthcare professions' remit will evolve due to the introduction of technological advancements (56). The disagreement by those with limited AI knowledge may also be explained due to a perceived loss of status and feelings of technical incompetence that is associated with the reorganisation of professional work following AI implementation (57). 'Status Loss' (*n* = 26) was within the top two themes of how AI implementation will potentially change radiographer roles and responsibilities (Table 6). Respondent R-4, (female, aged >35 years, intermediate AI knowledge), feels “*Radiographers will no longer be professionals but technicians or healthcare assistants*”.

Radiographers' roles will ultimately remain to serve the patients; a learned resilience with evolution. Also, those with advanced knowledge generally feel less vulnerable to change because they are more agile, and highly skilled jobs will be adapted to the evolving technological landscape (58, 59).

Surprisingly, 38% of radiographers reported no AI-specific education/training, whilst the majority that had (32.8%) were self-taught through personal reading. Consequently, 70.8% of respondents had no formal AI education/training. Only 29.2% of radiographers have received formal AI education/training with the majority being supplied by industry (8.1%). Although industry education is vital for the safe implementation of AI, it is essential that more higher education institutions (HEIs) start to offer easily accessible radiographer-specific AI training that is pedagogical and unbiased.

### 4.3 Optimism VS. pessimism

An overall sense of optimism was noted for the short-term (Figure 3), and medium-to-long-term (Figure 6) impacts of AI implementation on radiographer roles and responsibilities. The optimism was stronger for the long-term impact of AI (60.7%) in comparison to the short-term impact of AI (43.1%). This paints an optimistic viewpoint by radiographers for the future of their job roles and professional identity. However, the qualitative answers provide greater insights and potentially present a more diverse picture. There are notable concerns and mixed messages which need to be further analysed and explored. Concerns around AI implementation included its impact on professional deskilling and the often promised but infrequently realised efficiency of AI in clinical scenarios. Radiographer's over-reliance on AI and subsequent loss of autonomy were also of great concern. To mitigate these concerns, radiographers need more AI education and training to give them the confidence to understand AI-enabled decisions and override them when necessary. Radiographers also need to be more involved in developing and implementing clinical AI to ensure all deployed AI is trustworthy, clinically relevant, reliable, ethical and fit for purpose (34). Radiographer AI leadership/champion roles should be developed to oversee safe AI deployment, reassuring the radiographer workforce about the AI they are using (5, 60).

### 4.4 Common hopes and concerns amongst professionals

A few studies have surveyed radiologists' and radiographers' opinions about how they perceived AI would affect their profession, with mixed findings on whether AI would likely support their practice (5, 47, 61).

The results from this study reflect those of Huisman et al., who explored the attitude of radiologists and radiology residents worldwide towards implementing AI and the impact on their jobs. Fear of replacement was higher in those with limited AI knowledge, whereas those with advanced AI knowledge had more positive attitudes towards AI. In Huisman et al. (47), 79% of radiologists called for AI education to be incorporated in residency programs, just as this study calls for HEIs to increase the opportunities of AI education offered to radiographers, to increase trust and tackle fear of replacement.

This study has come three years after Rainey et al. (5), where radiographers expressed the need for more AI education as they considered themselves not adequately trained to implement or work with AI. It appears this is still the case, with 38% of all radiographers responding to this survey having no AI education and only 12% getting their AI education from a HEI (undergraduate, postgraduate and CPD combined). Career incentives, more choices on educational provisions about AI and endorsement by professional bodies will help these numbers improve with time. In a world first, the Health and Care Professions Council (HCPC), under the strong advocacy of the Society and College of Radiographers has now added digital competencies, including AI, as a core competency for registration (62).

In terms of job opportunities with AI implementation, radiologists believed there would be an increase in job opportunities with AI implementation (61), whereas this study found radiographers were much more unsure about future job opportunities. Radiographers currently lack active representation within the AI ecosystems, which might deprive them of the foresight of potential new job opportunities that may arise from the implementation of AI. Respondent R-263 (non-binary, aged >35 years, basic AI knowledge) voiced these concerns quoting “*I am concerned that decisions may be made about AI that affect radiographers without their involvement*” when asked what concerns them about the impact AI will have on radiography practice (Table 6). However, this is changing with multidisciplinary societies that bring together many different professionals in an organic way (63, 64) and with cross-disciplinary collaborations between existing organisations and different agencies in the UK and Europe (15).

This study, in agreement with previous literature (5, 15), shows that despite some reservations, there is a generally favourable attitude towards AI by UK radiographers. Their concerns may differ relative to their roles, experience and gender, but the overall feeling is that the majority of concerns can be overcome with additional training and customised education.

## 5 Limitations

The snowball sampling technique, despite enhancing recruitment, has the possibility of causing selection bias within the cohort of respondents. Subsequently, the population of respondents may have been less diverse, as radiographers working in remote regions or those without presence on social media may not have had access to an online survey (65).

This cross-sectional study can only give a snapshot view of radiographers' current perceptions. The AI landscape within healthcare is rapidly changing, and radiographers' perceptions of how AI may affect their roles and responsibilities will likely evolve.

## 6 Conclusion

UK-based radiographers are generally optimistic about AI deployment and future career opportunities. They feel they will have increased responsibilities and acknowledge the upcoming change that will affect all aspects of their professional identity. Their reservations relate primarily to the fear of deskilling, the increasing over-reliance and dependence on technology that might undermine their professional status and autonomy, as well as the excitement surrounding AI, where many promises for efficiencies have yet to materialise and are eagerly anticipated. The findings emphasise the importance of developing tailored educational programs and fostering AI leadership within the profession. These have the potential to mitigate fears of obsolescence and ensure radiographers are prepared to deliver the digital future. Consequently, trust and confidence are instilled in the process and the outcomes of AI implementation.

## Data Availability

The raw data supporting the conclusions of this article will be made available by the authors, without undue reservation.
