# Supplementary material for: R-AI-diographers: investigating the perceived impact of artificial intelligence on radiographers' careers, roles, and professional identity in the UK
Source: Front Digit Health. 2025 Dec 8;7:1603511. doi: 10.3389/fdgth.2025.1603511 (PMC12719279; doi:10.3389/fdgth.2025.1603511)
Supplement: Supplementary file 1 [file Datasheet1.pdf]

CITY
UNIVERSITY OF LONDON
EST 1894

Block 1

R-AI-diographers: Exploring the changing professional role and identity of radiographers in Europe in the era of artificial intelligence (AI)

Rationale of the study

Artificial intelligence (AI) is being increasingly applied in medical imaging and radiotherapy and has the potential to improve quality, safety and efficiency of care and clinical service provision in these areas of clinical practice. Radiographer responsibilities traditionally balance between patient care and imaging or radiation therapy technology.

AI has the potential to impact radiographers in many ways, including their professional roles, collective professional identity and career prospects and options.

The aim of this work is to explore the impact of AI implementation on both the student and qualified radiographers' roles, careers and professional identity in Europe.

This survey will gather experiences and perspectives of radiographers across Europe, with regards to the future of their profession considering AI developments in our professional space. The data will be used to offer recommendations that could help enhance patient care, academic and clinical training of radiographers, but also to address the mismatch between educational AI provisions and clinical practice and to support a sustainable future for radiography in Europe.

This project is a collaboration between City, University of London (School of Health and Psychological Sciences and the Bayes Business School), Vrije Universiteit Amsterdam, and University College Cork. It also has the endorsement of the Society and College of Radiographers (SCoR) and the European Federation of Radiographer Societies (EFRS). This research has been funded by the College of Radiographers Industry Partnership Scheme (CoRIPS).

Ethics approval has been granted by City, University of London (Ref ETH2223-1346).

Inclusion Criteria

Please complete the survey **if you meet all** the following inclusion criteria:
1. You are over 18 years of age,
2. You are a radiographer (student radiographer or any of the following: diagnostic, therapeutic, nuclear medicine technologist, or sonographer). Retired radiographers are also eligible to participate.
3. You work in the UK or Europe. This could involve working in a clinical setting, in higher education, research, industry or a radiography related policy job e.g. working in a professional body or a regulatory body.

Survey completion
It should take approximately **15 minutes to complete**. You can go back to change an answer, if required. You can come back later to finish off the survey if needed (within 24 hours).

As this survey is **anonymous** you cannot withdraw your data. Please click **"consent"** below if you would like to proceed. Many thanks for your time and for kindly sharing your perspectives.

○ I consent

Default Question Block

What is the gender you identify with? Please choose the one that applies or type accordingly in the final option.

- Female
- Male
- Non-binary
- Prefer not to say
- Other (please specify)

What is your current age in years? Please choose the one that applies from the given options.

- 18-25
- 26-35
- 36-45
- 46-55
- 56-65
- >65
- Prefer not to say

In which country do you currently practice (work) or study Radiography? Please select your answer from the dropdown options below.

What is currently your radiography experience in years? Please choose the one that applies from the given options (*collectively, your time spent as a student, clinical experience and radiography work outside of clinical e.g. academic work or research if applicable*).

- 0-2
- 3-5
- 6-10
- 11-20
- >20
- Not practicing
- Retired

What is your main current role? Please choose the ONE that applies from the given options.

- Undergraduate Student Radiographer

- ☐ Apprentice Radiographer
- ☐ Assistant Practitioner Radiographer
- ☐ Clinical Radiographer (please only choose this if none of the other clinical roles apply)
- ☐ Research Radiographer
- ☐ Advanced Practitioner Radiographer
- ☐ Consultant Radiographer
- ☐ Radiology Manager
- ☐ Clinical Academic- Practitioner + Teaching
- ☐ Academic in Radiography- Teaching Only
- ☐ Academic in Radiography- Teaching + Research
- ☐ PhD Student in Radiography
- ☐ Retired Radiographer
- ☐ Other (please specify)

What Radiography speciality are you trained/training in? Please choose the one that applies from the given options.

- ○ Diagnostic Radiographer
- ○ Therapeutic Radiographer
- ○ Both diagnostic and therapeutic radiographer
- ○ Nuclear Medicine Technologist
- ○ Sonographer

- Other (please specify)

What is your highest academic qualification ? Please choose the one that applies from the given options.

- I'm still studying for my undergraduate/bachelor's degree
- BSc (or DCR or equivalent)
- Post Graduate Certificate
- Post Graduate Diploma
- Master's (or MBA or equivalent)
- PhD/Professional Doctorate
- Other (please specify)

In what clinical setting do you perform most of your clinical work? Please choose the one that applies from the given options.

- Public Hospital
- Private Hospital/ centre
- Research centre/ Institute/ facility
- Mobile Unit

- Other (please specify)

- I do not work in a clinical setting

What is your knowledge on the **use of AI in Radiography**? Please choose the one that applies from the given options.

- Never heard of AI
- Basic Knowledge
- Intermediate Knowledge
- Advanced Knowledge
- Expert- I lead research in this field

What forms of education and training in AI have you participated in? Please choose all options that apply.

- [ ] None
- [ ] Undergraduate Level at University (please explain)
- [ ] Postgraduate Level at University (please explain)
- [ ] CPD Education at University (please explain)

- ☐ CPD Education by Professional Society (please explain)
- ☐ CPD Education by Company (please explain)
- ☐ Self-taught-personal reading
- ☐ Other (please specify)

What is your experience using AI in radiography? Please choose the one that applies from the given options.

- ◯ Never used AI to my knowledge
- ◯ Use occasionally
- ◯ Use daily
- ◯ Involved in research/development
- ◯ Other (please specify)

Please indicate your level of agreement with the following statement:
"*With the integration of AI in clinical practice, radiographers will be required to focus MOSTLY ON PATIENT CARE (consent, positioning, cannulation) and be less involved in technology*".

- Strongly Agree
- Agree
- Neither agree nor disagree
- Disagree
- Strongly Disagree

Please indicate your level of agreement with the following statement:
"*With the integration of AI in clinical practice, radiographers will be required to focus MOSTLY ON TECHNOLOGY (innovation, optimisation, quality control) and less on patient-related responsibilities*".

- Strongly Agree
- Agree
- Neither agree nor disagree
- Disagree
- Strongly Disagree

Please indicate your level of agreement with the following statement:
"*With the integration of AI in clinical practice, radiographers will have MORE TIME to spend with patients due to AI advancements and faster workflow*".

- Strongly Agree

- Agree
- Neither agree nor disagree
- Disagree
- Strongly Disagree

Please indicate your level of agreement with the following statement:
"With the integration of AI in clinical practice, radiographers will have LESS TIME to spend with patients due to AI advancements and a potential increase in workload".

- Strongly Agree
- Agree
- Neither agree nor disagree
- Disagree
- Strongly Disagree

Please indicate your level of agreement with the following statement:
"Despite the advancement of AI, image quality and treatment quality will remain the responsibility of the radiographers".

- Strongly Agree
- Agree

- Neither agree nor disagree
- Disagree
- Strongly Disagree

Please indicate your level of agreement with the following statement:
"*Medical image and treatment quality will become the responsibility of AI (with appropriate quality assurance checks)*".

- Strongly Agree
- Agree
- Neither agree nor disagree
- Disagree
- Strongly Disagree

With regards to AI advancements, radiographer **technology-related problem-solving skills** (e.g. quality assurance or optimisation of imaging/treatment parameters to suit patient anatomy and pathology) will:

- Remain the same
- Increase
- Decrease

○ Other (please specify)

With regards to AI advancements, radiographer **patient-centred skills** (e.g.adaptive techniques and communication to address patient needs) will:

○ Remain the same
○ Increase
○ Decrease
○ Other (please specify)

With regards to AI advancements, radiographer **radiation protection responsibilities** will:

○ Remain the same
○ Increase
○ Decrease
○ Other (please specify)

With regards to AI advancements, radiographer **job and career opportunities** will:

- ○ Remain the same
- ○ Increase
- ○ Decrease
- ○ Other (please specify)

Please indicate your level of agreement with the following statement:
*"With regards to AI advancements, radiographers will need to work **closer with the patients**".*

- ○ Strongly Agree
- ○ Agree
- ○ Neither agree nor disagree
- ○ Disagree
- ○ Strongly disagree

Please indicate your level of agreement with the following statement:

"With regards to AI advancements, radiographers will need to work ***closer with other medical imaging and radiotherapy professionals****".*

- Strongly Agree
- Agree
- Neither agree nor disagree
- Disagree
- Strongly disagree

Please indicate your level of agreement with the following statement:
"AI will only ever assist radiographers, never replace them".

- Strongly Agree
- Agree
- Neither agree nor disagree
- Disagree
- Strongly Disagree

Please indicate your level of agreement with the following statement:
"With time, AI will ultimately replace radiographers".

- Strongly Agree
- Agree

- Neither agree nor disagree
- Disagree
- Strongly Disagree

Please indicate your level of agreement with the following statement:
"Radiographers will evolve with AI, and roles and professional ***identity may be quite different*** *from today".*

- Strongly Agree
- Agree
- Neither agree nor disagree
- Disagree
- Strongly Disagree

Please indicate your level of agreement with the following statement:
"Radiographers will be involved ***more in research and development*** *than in their current role(s)" .*

- Strongly Agree
- Agree
- Neither agree nor disagree
- Disagree
- Strongly Disagree

The following few questions are open-ended, so please feel free to answer them by adding some text to help us understand a bit more about your views. Thank you!

What are you **looking forward to** as AI comes into daily radiographer practice? Please explain your answer(s).

What **concerns you more** as AI comes into radiography practice? Please explain you answer(s).

How do you think radiographer **roles and responsibilities will change**, if at all, with the introduction of AI?

What do you expect the **impact of AI** on radiographer professional autonomy will be?

What do you feel **you mostly need** to be better prepared to work with AI in your practice?

Are Radiographers well prepared **to lead the implementation of AI** in healthcare? Please explain your answer in the text box provided.

○ Yes (please explain)

○ No (please explain)

What are some day-to-day responsibilities you think an **AI Radiographer leader** could take on?

Would you feel confident to lead in an AI-enabled work environment? Please explain your answer in the text box provided

○ Yes (please explain)

○ No (please explain)

If you feel confident leading in an AI-enabled work environment, **what would motivate you** to consider an AI leadership role?

○ Please explain

○ I do not feel confident leading in an AI enabled environment

Block 3

Thank you for taking the time to complete this survey.

If you are happy to be contacted regarding participation in future projects in this topic area, please leave your details below (but this would remove your anonymity).

☐ Name:

☐ Email Address:

☐ I do not wish to be contacted further

Powered by Qualtrics
